# ProT‐Diff: A Modularized and Efficient Strategy for De Novo Generation of Antimicrobial Peptide Sequences by Integrating Protein Language and Diffusion Models

**DOI:** 10.1002/advs.202406305

**Published:** 2024-09-25

**Authors:** Xue‐Fei Wang, Jing‐Ya Tang, Jing Sun, Sonam Dorje, Tian‐Qi Sun, Bo Peng, Xu‐Wo Ji, Zhe Li, Xian‐En Zhang, Dian‐Bing Wang

**Affiliations:** ^1^ Key Laboratory of Biomacromolecules (CAS), National Laboratory of Biomacromolecules, CAS Center for Excellence in Biomacromolecules, Institute of Biophysics Chinese Academy of Sciences Beijing 100101 China; ^2^ Precision Scientific (Beijing) Co. Ltd. Beijing 100085 China; ^3^ Faculty of Synthetic Biology Shenzhen Institute of Advances Technology Shenzhen 518055 China; ^4^ University of Chinese Academy of Science Beijing 100049 China; ^5^ Department of Biotechnology, School of Life Sciences Shandong Normal University Jinan 250014 China

**Keywords:** antimicrobial peptide, artificial intelligent, de novo design, diffusion model, protein language model

## Abstract

Antimicrobial peptides (AMPs) are a promising solution for treating antibiotic‐resistant pathogens. However, efficient generation of diverse AMPs without prior knowledge of peptide structures or sequence alignments remains a challenge. Here, ProT‐Diff is introduced, a modularized deep generative approach that combines a pretrained protein language model with a diffusion model for the de novo generation of AMPs sequences. ProT‐Diff generates thousands of AMPs with diverse lengths and structures within a few hours. After silico physicochemical screening, 45 peptides are selected for experimental validation. Forty‐four peptides showed antimicrobial activity against both gram‐positive or gram‐negative bacteria. Among broad‐spectrum peptides, AMP_2 exhibited potent antimicrobial activity, low hemolysis, and minimal cytotoxicity. An in vivo assessment demonstrated its effectiveness against a drug‐resistant *E. coli* strain in acute peritonitis. This study not only introduces a viable and user‐friendly strategy for de novo generation of antimicrobial peptides, but also provides potential antimicrobial drug candidates with excellent activity. It is believed that this study will facilitate the development of other peptide‐based drug candidates in the future, as well as proteins with tailored characteristics.

## Graphical abstract

ProT‐Diff integrates pretrained protein language and diffusion models for the de novo generation of antimicrobial peptides. Of the 45 peptides selected, 44 demonstrated antimicrobial activity, with AMP_2 exhibiting potent broad‐spectrum efficacy, minimal cytotoxicity, and in vivo effectiveness against drug‐resistant *E. coli*. This study paves the way for the development of peptide‐based drug candidates and proteins with tailored characteristics.

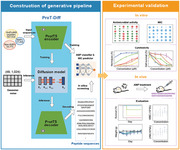

## 1 Introduction

The emergence of antibiotic‐resistant pathogens has become a public health concern that poses a considerable threat to human health. However, since the 1990s, the pace of development and commercialization of new antibiotics has declined, with only a limited number of antimicrobial therapies receiving approval from regulatory agencies, such as the U.S. Food and Drug Administration and the European Medicines Agency.^[^ ^1^ ^]^ Therefore, developing efficient methods for identifying alternative molecules to combat drug resistance is imperative.

Antimicrobial peptides (AMPs), a diverse group of peptides found across all life forms, play a vital role in the innate immune response by eradicating various pathogens, including antibiotic‐resistant microbes.^[^ ^2^, ^3^ ^]^ These peptides eliminate pathogens through diverse mechanisms, including membrane disruption, DNA degradation, and hindering oxygen uptake.^[^ ^4^, ^5^, ^6^ ^]^ Furthermore, because of the reduced risk of fostering antimicrobial resistance, AMPs have garnered substantial attention as the next‐generation antibiotics.^[^ ^7^, ^8^, ^9^ ^]^ Over the past few decades, numerous AMPs have been identified from natural sources, primarily through experiments, with some progress in clinical trials.^[^ ^10^ ^]^ However, limitations regarding their efficacy, stability, and toxicity persist, hindering their broad clinical applicability. Consequently, several advanced high‐throughput techniques have been developed to discover new AMPs with enhanced performance.^[^ ^11^, ^12^ ^]^ However, these experiment‐driven approaches are time‐consuming and costly, which limits the efficiency of searching for desired drug candidates.

Recent advances in artificial intelligence‐based approaches have facilitated AMPs discovery. For instance, a predictive pipeline that integrates multiple natural language processing neural network models was developed to discriminate AMPs in the human gut microbiome.^[^ ^13^ ^]^ This approach efficiently explores vast sequence datasets, thereby reducing time required for mining novel functional peptides but still faces challenges in generating peptide sequences that do not exist in nature. To address this limitation, a unified pipeline was used to identify three unnatural hexapeptides with excellent antibacterial activity by extensively exploring the sequence space within a pre‐constructed dataset of six to nine amino acids.^[^ ^14^ ^]^ However, this pipeline must utilize a pre‐constructed database, which may considerably limit the explored search space, thereby restricting the length of the generated AMPs. Moreover, deep generative neural networks, such as Variational Autoencoders, Long Short‐Term Memory, Generative Adversarial Network, and their variants, have demonstrated their capacity to generate functional AMPs from scratch with wet‐lab experimental validation.^[^ ^15^, ^16^, ^17^, ^18^, ^19^, ^20^ ^]^ However, these models couple the processes of sequence representation and generation, resulting in limitations on the length, diversity, and success rate of the generated sequences.

Considering these limitations, developing a novel, user‐friendly pipeline that can rapidly and efficiently generate abundant unnatural AMPs without requiring prior knowledge of peptide structures or amino acid sequence alignments is necessary. Therefore, we developed an innovative integration of pretrained protein language models with a class of emerging generative models, specifically diffusion models, to design AMPs. Pretrained large protein language models have demonstrated their proficiency in uncovering the intricate syntax of protein sequences and extracting features from such sequences.^[^ ^21^, ^22^, ^23^, ^24^, ^25^, ^26^ ^]^ Diffusion models represent the zenith of generative capabilities and are excellent for the controllable generation of images and texts.^[^ ^27^, ^28^, ^29^, ^30^, ^31^, ^32^, ^33^ ^]^ By decoupling the tasks of sequence representation and generation, we leveraged the inherent strengths of both models, captured informative patterns using a protein language model, and generated novel AMPs sequences by applying diffusion models to reconstruct the latent features.

Building on the aforementioned idea, we developed a novel and modularized deep generative model strategy, ProT‐Diff, by integrating a diffusion model between the decoder and encoder of a pretrained language model. Our ProT‐Diff model was effective in generating unnatural AMPs with markable diversity and a wide range of amino acids. After in silico screening of thousands of generated AMPs based on their physicochemical properties and predicted antimicrobial activities, we synthesized and experimentally evaluated 45 generated candidate AMPs, and found that 44 displayed efficacy against either gram‐positive or gram‐negative bacteria. We assessed a promising AMP candidate for in vivo antimicrobial activity in a mouse model of acute peritonitis. We believe that this study is crucial for developing functional peptide‐based drugs through AI‐model‐driven sequence design.

## 2 Results

### 2.1 Development of a Deep Generative Model for AMPs

**Figure 1:**
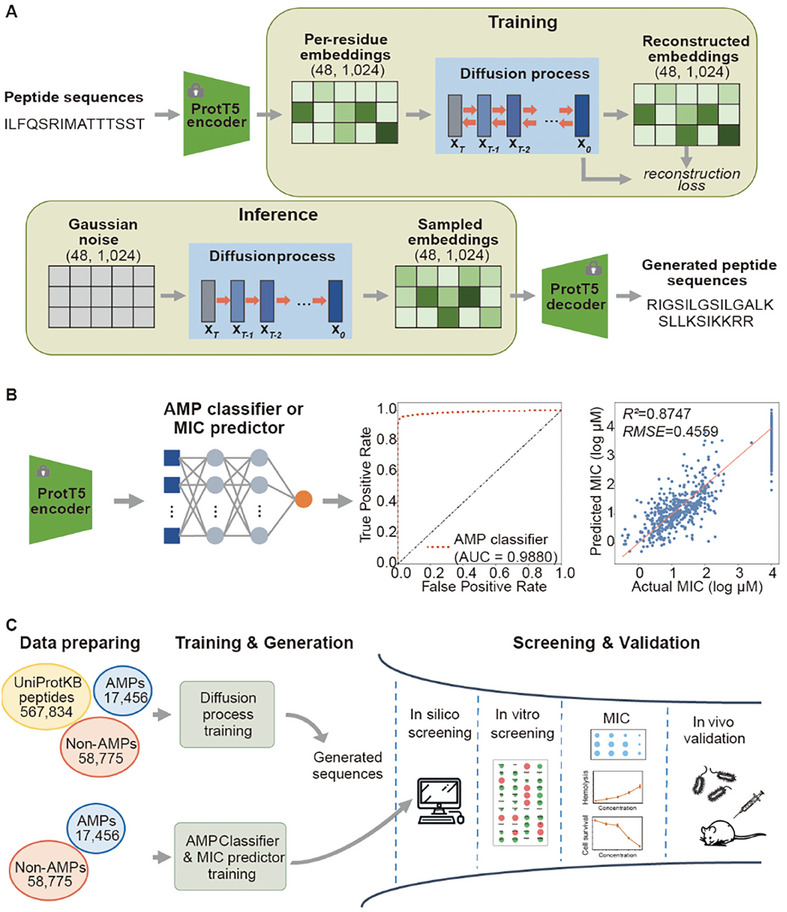
Overview of the generative pipeline for AMPs. A)The model architecture of ProT‐Diff. ProT‐Diff involves a decoupled and frozen pretrained encoder and decoder of ProtT5‐XL‐UniRef50. Positioned in the middle is a diffusion model that operates on a continuous space with dimensions of (48, 1024). This model is trained on custom peptide datasets. During training, the ProtT5 encoder embeds the input peptide sequences into the latent space, while the diffusion model is trained in a self‐supervised manner to reconstruct the peptide embeddings. In the inference phase, the diffusion model denoises the initial Gaussian latent feature to generate novel peptide embeddings, which are subsequently decoded by the ProtT5 decoder into peptide sequences. B) The model architecture of the AMP classifier and MIC predictor, the Receiver Operating Characteristic (ROC) curve of the AMP classifier, and the correlation between the actual log MIC value and the predicted log MIC value by the MIC predictor on the test set. RMSE, root mean square error. n = 1815. C) Workflow for generating and screening candidate AMPs.Novel peptides are generated utilizing the trained diffusion model. An AMP classifier and MIC predictor are trained in parallel. To refine the generated peptides, a series of in silico filters are applied, and the resulting candidate peptides are synthesized and subjected to both in vitro and in vivo validation.

To address the challenges of developing AMPs through de novo design, we introduced a deep generative model that sandwiches a continuous diffusion model between the encoder and decoder of the transformer‐based protein language model ProtT5‐XL‐UniRef50.^[^ ^26^ ^]^ This combination enabled us to benefit from ProtT5's robust feature extraction capabilities while harnessing the diffusion model's ability to generate continuous tensors (**Figure** **1**A). The ProtT5 encoder and decoder were decoupled to facilitate peptide sequence manipulation. This decoupling enabled us to project peptide sequences onto a continuous latent space using the ProtT5 encoder and then translate the generated tensors back into peptide sequences using the ProtT5 decoder. The parameters of both the encoder and the decoder can be frozen without further finetuning. To construct a generative pipeline for peptides with specific properties, such as AMPs, we only need to train a continuous diffusion model in the latent space using a well‐defined peptide dataset that exhibits the desired properties. This approach maximizes the efficacy of the pretrained protein language model and minimizes the GPU memory, training time, and training data requirements.

To handle the variability in the length of the input peptides, we padded the per‐residue embeddings derived from the ProtT5 encoder to a fixed shape of (48, 1024), with zeros. The diffusion model was configured to have a fixed shape. The original peptide features generated by the diffusion model mirrored the shapes (48, 1024) of the input padded peptide embeddings. Because these generated tensors preserved the padding patterns of the input peptide features, we removed rows that primarily comprised values close to zero. This truncation process enhanced the decoding of these features into peptide sequences and simplified the reconstruction of peptides of varying lengths.

During the training phase, the diffusion denoising network takes the padded peptide embeddings as input and recovers the input embeddings step‐by‐step in a self‐supervised manner. In the inference phase, the latent variables were initially sampled from a Gaussian distribution and subsequently processed through the reverse steps of the trained diffusion model to obtain denoised peptide embeddings in the ProtT5 latent space (Figure 1A).

### 2.2 Efficient Generation of Candidate AMPs

To generate and screen candidate AMPs, we acquired known AMPs from publicly available AMP databases: Collection of Anti‐Microbial Peptides (CAMPR4),^[^ ^34^ ^]^ A Database of Anti‐Microbial Peptides (ADAM),^[^ ^35^ ^]^ the Antimicrobial Peptide Database (APD3),^[^ ^36^ ^]^ and Giant Repository of AMP Activities (GRAMPA).^[^ ^37^ ^]^ In total 17456 known AMPs were collected after filtering based on the following criteria: 1) labeled as antibacterial, antifungal, antiviral, antimicrobial, and experimental validated; 2) have a length of 5–48 amino acids; 3) only contains capital letters, excluding “U, Z, O, B, and J” residues. The UniProtKB peptide dataset includes substrings randomly sampled from proteins present in the UniProtKB‐reviewed protein database. The length distribution of this dataset matches that of the known AMPs. In total, 567834 peptide sequences remained after de‐duplication. A non‐AMP dataset consisting of 58775 peptides was obtained from a previously published dataset.^[^ ^13^ ^]^

We employed two strategies during the training procedure: i) train the diffusion model with a combined dataset of the known AMPs and non‐AMPs, and ii) the pretrain‐finetune approach, in which we initially pretrained the diffusion model on the UniProtKB peptide dataset to learn a general syntax of protein sequences, and then finetuned the pretrained model on the specific AMP dataset to capture the distinctive features of AMPs. With a train‐test split ratio of 8:2, the entire training process of the diffusion model was completed in <40 h using a single RTX4070Ti GPU. Sampling of 500 peptide embeddings using the trained diffusion model required less than 5 min, while decoding 500 peptide embeddings to amino acid sequences required ≈30 min.

In parallel, we employed a three‐layer multi‐layer perceptron (MLP) architecture to train an AMP classifier and AMP minimal inhibitory concentration (MIC) predictor using labeled data. These components served as filters for the generated peptides (Figure 1B). We utilized the Area Under the Receiver Operating Characteristic (AUROC) curve to assess the performance of the AMP classifier and the coefficient of determination (R^2^) to evaluate the goodness of fit of the AMP MIC predictor. The trained AMP classifier achieved an AUROC of 0.988 for the test sets, demonstrating an excellent performance. The MIC predictor yielded an R^2^ value of 0.875, indicating a high level of accuracy. Our AMP classifier and MIC predictor outperformed most previous models, indicating that the pretrained protein language model is well‐suited for AMP encoding and is a cornerstone of downstream tasks.^[^ ^38^ ^]^

We initiated the generation process by sampling random variables from the Gaussian distributions. In cases in which the diffusion model was trained on a single dataset with a limited number of AMPs and non‐AMPs, we introduced uniform noise distributions instead of the original Gaussian noise at each time step to enhance the diversity of the generated content. Increasing the dispersion of the sampled noise generated more diverse tensors (Figure S1, Supporting Information). In addition, in the pretraining and finetuning scenarios, where the diffusion model was fed with ample training data, the default Gaussian noise was maintained, resulting in adequate generation diversity.

To generate a sufficient number of peptides, we performed multiple iterations of the sampling procedure using different random seeds. The generated peptides were fed into a series of in silico filters. First, we removed duplicate peptides and those that were already present in the AMP dataset. Subsequently, we retained only peptides that were predicted to be AMPs by our trained classifier. Based on empirical observations, 99.35% of known AMPs, whether natural, synthetic, or predicted, and experimentally validated in public databases, contain a maximum of six consecutive amino acids in tandem repeats (e.g., CCCCCC). Additionally, positively charged AMPs have a higher propensity to interact with negatively charged surfaces of bacterial membranes.^[^ ^39^, ^40^ ^]^ A highly positive charge is also associated with an elevated risk of hemolytic activity and cytotoxicity.^[^ ^7^, ^37^ ^]^ Therefore, we imposed several constraints on the peptides, including a maximum of 6 tandem repeats of amino acids, a positive charge requirement, and a restriction of ≤40% residues being either arginine (R) or lysine (K).^[^ ^37^ ^]^ Subsequently, the generated peptides were subjected to i) in silico filters for high‐confidence candidate AMPs, ii) in vitro validation, and iii) in vivo validation (Figure 1C).

### 2.3 In Silico Assessment of Candidate AMPs

**Figure 2:**
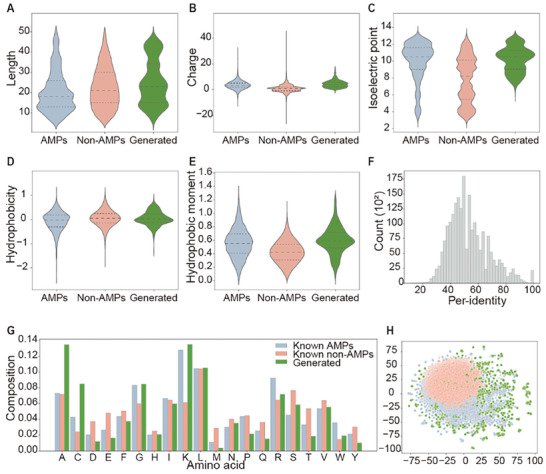
Physicochemical properties of the generated AMP candidates post in silico filtering. A) The amino acid lengths of peptide sequences. B) The theoretical net charge (pK scale = Dawson). C) The isoelectric point (pK scale = Dawson). D) The hydrophobicity index (scale = Eisenberg). E) The hydrophobic moment. F) Per‐identity of the generated candidate peptides to known AMP dataset calculated by BLASTP. G) Amino acid composition. H) t‐SNE projection of peptide embeddings. Blue dots represent known AMPs, red dots represent known non‐AMPs, and green dots represent generated set. n = 17456 for AMPs, n = 58775 for non‐AMPs, and n = 3133 for generated set.

Next, we conducted an in silico analysis to examine the physicochemical properties of the selected candidate AMPs (**Figure** **2**). The analysis revealed that most of the generated peptides had lengths ranging from 10 to 25 amino acids, which is consistent with the length distribution observed in known AMPs (Figure 2A). In addition, the filtered generated peptides displayed elevated values of net charge, isoelectric point, and hydrophobic moment compared to the non‐AMP dataset (Figure 2B–E). They also demonstrated an amino acid composition analogous to that of known AMPs (Figure 2G), suggesting that the trained diffusion model effectively captured the essential physicochemical properties of known AMPs that were manifested in the generated peptide set. To assess the sequence identity between known AMPs and generated AMPs, we conducted a sequence search using BLASTP. Sequence identities ranged from <20% to 100% (Figure 2F), indicating that the diffusion model could replicate the sequences present in the training set and generate completely novel sequences. Moreover, t‐SNE projections demonstrated the dual functionality of the model in generating both known and novel sequences. The projections of the embeddings derived from the filtered generated peptides clustered with the padded peptide embeddings of known AMPs in the training set, while exhibiting a wider distribution than the known AMPs (Figure 2H). Collectively, these results suggested that this model can generate highly effective AMPs that do not naturally occur in biological systems.

### 2.4 In Vitro Evaluation of Antimicrobial Activity for Candidate AMPs

**Figure 3:**
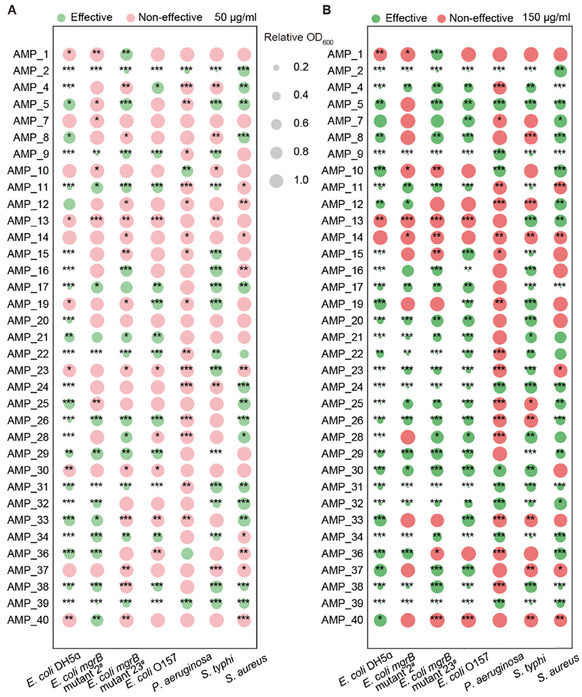
In vitro evaluation of antimicrobial activity for generated AMP candidates. The antimicrobial activities of the 35 synthesized candidate AMPs were assessed at concentrations of 50 µg mL^−1^ A) and 150 µg mL^−1^ B) against multiple bacteria in liquid medium. The relative OD_600_ of the experimental and control groups were compared, with an effectiveness threshold of 0.8. Any relative OD_600_ value below 0.8 was considered effective. The means between the experimental and control groups were compared using a two‐sided Student's *t*‐test (“^*^” indicates 0.01 < *P* ≤ 0.05, “^**^” indicates 0.001 < *P* ≤ 0.01, and “^***^” indicates *P* ≤ 0.001). n = 4 for each group.

**Figure 4:**
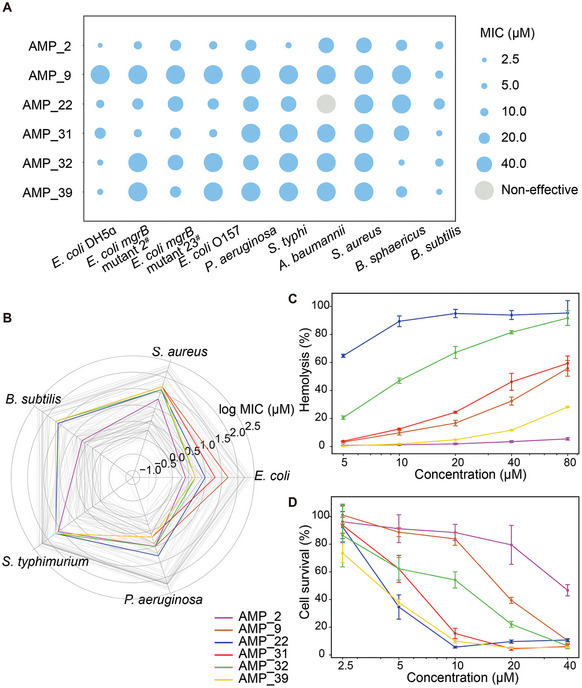
Minimum Inhibitory Concentration (MIC) and safety evaluation of generated broad‐spectr**um AMPs**. A) MIC values (µM) of the selected peptides against different bacteria are indicated by the size of the dots. MIC ≥ 60 µMis regarded as non‐effective and colored grey. B) Comparison between the log MIC values of the generated AMPs (colored) and the recorded log MIC values of well‐documented AMPs obtained from databases (grey, n = 117). C) Assessment of hemolysis of the generated AMPs. The curve shows the percentage of hemolysis with different concentrations (5–80 µm) of AMPs. Error bars indicate standard deviation of the mean, n = 3. D) Assessment of cytotoxicity of the generated AMPs. The curve shows the percentage of cell survival with different concentrations (2.5–40 µm) of AMPs. Error bars indicate standard deviation of the mean, n = 6.

To evaluate the performance of our predicted AMPs, we first selected 40 sequences with predicted MIC values below 10 µm for further chemical synthesis and experimental validation (Table S1, Supporting Information). These AMP candidates showed relatively high sequence identity with a known AMP dataset calculated using BLAST, of which 35 were successfully produced. We evaluated the antimicrobial activities of 35 selected peptides against *Escherichia coli*, *Pseudomonas aeruginosa*, *Salmonella typhi*, and *Staphylococcus aureus*. The antimicrobial activities were assessed by comparing the relative changes in OD_600_ between the test and control groups, with a threshold of 0.8 to differentiate between effective and non‐effective AMPs. Strikingly, >85% (30 out of 35) of the candidate AMPs effectively inhibited at least one bacterial strain at low concentrations and >97% (34 out of 35) exhibited antimicrobial activity at high concentrations (**Figure** **3**). We further selected 10 sequences with relatively low sequence identity, and all the tested sequences showed antimicrobial activity, with four demonstrating broad‐spectrum activity (Figure S2, Supporting Information). Notably, most of the tested AMPs displayed greater antimicrobial efficacy against *E. coli* than against other species, presumably because of the higher prevalence of AMPs targeting *E. coli* in the training set (Figure S3, Supporting Information). Given our primary objective of generating novel broad‐spectrum AMPs, we selected six synthesized AMPs (AMP_2, AMP_9, AMP_22, AMP_31, AMP_32, and AMP_39) and determined their minimum inhibitory concentration (MIC) (Figure 4). In addition to the bacteria used in the initial screening, we included two additional gram‐positive strains, *Bacillus sphaericus* and *Bacillus subtilis*, and one extra‐gram‐negative strain, *Acinetobacter baumannii*, in the MIC tests. The selected AMPs exhibited no discernible preference between gram‐negative and gram‐positive strains, as they demonstrated broad‐spectrum activity against all tested bacteria (**Figure** **4**A). All the selected AMPs consistently displayed higher MIC values (≥40 µM) when tested against *A. baumannii* and *S. aureus* compared to the other bacteria examined in this study. To verify the potential advantages of our newly developed AMP, we compared the selected AMPs with well‐documented AMPs obtained from databases based on their recorded MIC values. Interestingly, no existing records document AMPs with MIC values against all tested bacteria in our panel. Considering that 117 known AMPs with MIC were available against all tested bacteria, excluding *A. baumannii*, we compared this specific subset with our AMPs. Our AMP showed MIC values within a moderate range, devoid of distinct favorability or unfavorability (Figure 4B).

To assess the safety of the selected candidate AMPs, we evaluated their hemolytic activity and cytotoxicity. Hemolytic activity was determined using red blood cells (RBCs) from rabbits, whereas cytotoxicity against human cells was assessed using Cell Counting Kit‐8 (CCK‐8). Among the six AMP candidates, AMP_2 exhibited significantly lower hemolytic activity and cytotoxicity than the other AMPs (Figure 4C,D). Even at a concentration of 80 µm, AMP_2 did not induce noticeable hemolysis. In addition, at a concentration of 40 µM, AMP_2 maintained a cell survival rate of over 60%, signifying a half maximal inhibitory concentration (IC50) of AMP_2 higher than 40 µm. Considering that the MIC values of AMP_2 against most of the tested bacteria were around or below 20 µM, AMP_2 exhibited low toxicity at its MIC value.

### 2.5 In Vivo Evaluation of a Promising, Competent Broad‐Spectrum Candidate, AMP_2

**Figure 5:**
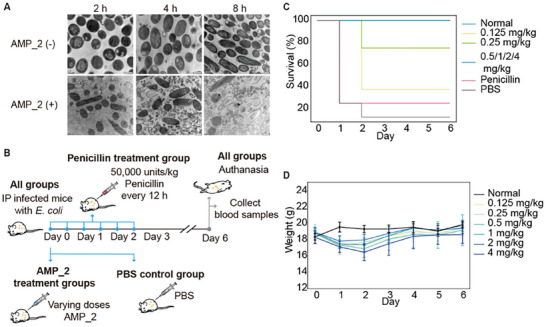
Antimicrobial mechanism and in vivo evaluation of antimicrobial activity of AMP_2. A) Electron microscopy images of *E.coli* DH5α treated without and with AMP_2 (1× MIC) at different time points. Scale bars: 500 nm. B) The workflow for evaluation of the efficacy of AMP_2 in a lethal mouse model of acute peritonitis. C) Kaplan‐Meier curves showing the survival of the mice with different treatment. D) Monitoring the body weight of surviving mice after treatment with various concentrations of AMPs in infected mice. Error bars indicate standard deviation of the mean.

As AMP_2 exhibited broad‐spectrum antimicrobial activity (low MIC value of 2.5 µm against *E. coli*.) coupled with its low hemolytic activity and cytotoxicity, AMP_2 appears to be a promising and competent broad‐spectrum AMP candidate. Therefore, we focused on this candidate for further evaluation and investigation into its antimicrobial mechanism. Since disrupting cell membrane integrity is a recognized antimicrobial mechanism, we utilized Transmission Electron Microscopy (TEM) to observe changes in bacterial morphology after treating *E. coli* DH5α with AMP_2 at its MIC for 2–8 h. AMP_2 induced noticeable morphological changes in the bacteria, leading to a serious leakage of cellular contents (**Figure** **5**A). In contrast, the untreated bacterial controls exhibited no apparent membrane permeability. Therefore, similar to known AMPs,^[^ ^41^ ^]^ the mechanism of action of AMP_2 involves integration into the bacterial cell membrane, thereby disrupting membrane integrity and culminating in cell lysis.

The intraperitoneal (IP) route of drug administration in laboratory animals is widely employed in numerous in vivo studies of disease models and has been substantiated as a justifiable route for pharmacological and proof‐of‐concept investigations.^[^ ^42^ ^]^ Therefore, we evaluated the antimicrobial activity of AMP_2 in a well‐known lethal mouse model of acute peritonitis, administered via IP.

To establish the animal model, we used different concentrations of the clinically isolated strain *E. coli mgrB* mutant 23^#^ to infect mice via IP injection (Figure 5B). This strain displayed multidrug resistance to a spectrum of antibiotics, including penicillin (data not shown). We found that a bacterial concentration of 5 × 10^5^ CFU mL^−1^ (CFU stands for colony‐formming unit) resulted in over 90% mortality within 24 h post‐infection, confirming the successful establishment of disease models, in line with previous reports.^[^ ^43^, ^44^, ^45^ ^]^ Following infection, the mice were IP administered various doses of AMP_2. Given the penicillin‐resistant nature of the *E. coli* strain, infected mice were treated with penicillin as a control. A single dose of 50000 units per kg was administered. While mice received a single treatment of AMP_2, antibiotic treatment was administered six times at 12‐h intervals over three days. Interestingly, even at the minimal dosage of 0.5 mg kg^−1^, AMP_2 conferred complete protection to *E. coli*‐infected mice (Figure 5C). In contrast, in the PBS treatment group, all deaths occurred within 24 h after the bacterial challenge, whereas in the other groups, deaths were observed within 48 h. According to the log‐rank test, there were significant differences in survival between the AMP_2 treatment group and the PBS treatment group (*P* < 0.05), whereas there was no significant difference between the penicillin treatment group and the PBS group (*P* > 0.05). Most surviving mice exhibited improved mental and physical well‐being within 24 h of treatment, as evidenced by increased activity and alertness. These mice were capable of consuming food and water normally, and their fur appeared soft and glossy. Monitoring the body weight of the mice revealed a consistent increase for the surviving mice treated with AMP_2 starting from the second‐day post‐infection (Figure 5D). By the sixth day post‐infection, while the 4 mg kg^−1^ treatment still resulted in a slight decrease in body weight, the mice in the other AMP_2 treatment groups had nearly restored their body weight to that of normal mice. Furthermore, routine blood tests revealed that many routine blood components in the treatment group were not significantly different from those in the control group (Figure S4, Supporting Information). Collectively, AMP_2 demonstrated promising therapeutic effects and a satisfactory safety profile in the murine model of lethal acute peritonitis.

## 3 Discussion

In this study, we developed a deep generative approach called ProT‐Diff, which combines a protein language model and a diffusion model to generate AMPs from scratch. By decoupling the tasks of sequence representation and generation, our approach overcomes previous limitations, especially in terms of success rate, enabling the effective and automated generation of novel AMPs. Validation of the 45 selected AMP candidates revealed that 44 of them exhibited antimicrobial activity, highlighting the high accuracy of our approach.

ProT‐Diff has several advantages over previous AMPs discovery models (Table S3, Supporting Information). First, the pretrained protein language model ProtT5‐XL‐UniRef50^[^ ^26^ ^]^ in ProT‐Diff presented strong ability to extract representations that reflect AMP attributes. Despite the low sequence similarity and high structural diversity among known AMPs, there was a clear clustering of natural AMPs and non‐AMPs in the t‐SNE projections of the peptide embeddings (Figure 2H). These findings, along with the high accuracy demonstrated by the AMP classifier and MIC predictor (Figure 1B), indicate the direct production of meaningful semantic representations of protein sequences by the pretrained language model without any finetuning. The excellent performance in extracting representations not only eliminates the need for prior knowledge of structures and sequence alignments but also allows our proposed strategy to be easily applied to various peptide datasets and efficiently processed on consumer‐grade GPUs.

Second, our ProT‐Diff diffusion model strikes a balance between diversity and fidelity in peptide sequence design, even with training sets comprising only a few thousand AMPs. Diffusion models have been proven to exhibit excellent performance in generating novel and high‐quality data, particularly those with high dimensionality or complex structures.^[^ ^46^ ^]^ The significant fidelity produced by the diffusion model was also achieved in our study, as the length, amino acid composition, physicochemical properties, and predicted structures, along with the clustering of the peptide embeddings in t‐SNE projections, presented similar distributions between the generated set and the known AMPs set (Figure 2). To overcome the challenge of maintaining diversity in generating products when the training data are limited, we optimized the noise distributions during sampling from the diffusion model or pretrain the diffusion model, followed by finetuning. This approach was further supported by the observation that the majority of uniquely generated sequences were not present in the training set (Table S2, Supporting Information) and that the lowest sequence identity between the generated and training sequences was <30% (Figure 2F). Furthermore, compared to training directly on a single peptide dataset, pretraining and finetuning of the diffusion model led to even greater generation diversity and fidelity (Table S2, Supporting Information).

Third, our modularized framework combines the strengths of the language and diffusion models, enhancing the generative capabilities of language models while reinforcing the representation capabilities of diffusion models. Conventional diffusion models typically operate on continuous data, whereas peptide sequence generation involves discrete data. Hence, the diffusion model can operate and leverage its advantages by utilizing only a protein language model to map discrete amino acid sequences to a high‐dimensional latent space. Additionally, because the language model can naturally process variable‐length sequences and the diffusion model excels in reconstructing the trained data pattern, ProT‐Diff can generate AMPs with a wide length range. Considering the high cost and difficulty associated with the solid‐phase chemical synthesis of peptides of >50 amino acids, we set a maximum length of 48 amino acids in our study. Consequently, ProT‐Diff enabled the generation of AMPs up to 48 amino acids in length. In contrast, previous AMP generation models that did not incorporate language models generated relatively short AMPs (<25 amino acids) or peptides of fixed length.^[^ ^15^, ^17^, ^47^ ^]^

To explore the structure of the chosen candidate AMPs, we employed AlphaFold2^[^ ^48^ ^]^ and SABLE^[^ ^49^ ^]^ tools to predict the secondary and tertiary structures. The majority of the predicted structures (35 out of 50) exhibit amphiphilic (hydrophilic and hydrophobic) α‐helical structures (Table S1, Supporting Information), which are widely observed in natural AMPs.^[^ ^50^ ^]^ Furthermore, the predicted structures comprised αβ structures, β‐strand structures and non‐αβ structures, highlighting the diversity of subtypes among the generated peptides (Figure S5, Supporting Information).

Among the selected AMPs with broad‐spectrum efficacy, AMP_2 demonstrated effectiveness against various drug‐resistant bacteria with low MIC values and high safety. In general, a higher antimicrobial potency in antimicrobial peptides often correlates with an elevated risk of heightened cytotoxicity and hemolytic activity. For example, melittin, a well‐known natural AMP, shows exceptional antimicrobial activity but also exhibits high hemolysis and cytotoxicity. The MIC of melittin against *E. coli* falls within the range of 1–15 µm, comparable to our AMP_2.^[^ ^51^, ^52^, ^53^ ^]^ However, even at a low concentration of 10 µg mL^−1^ (3.5 µM), melittin induces >50% human cell death,^[^ ^54^ ^]^ and at 1 µg mL^−1^ (0.35 µM), it causes 50% hemolysis of cells.^[^ ^55^ ^]^ In comparison, AMP_2 exhibited a more favorable hemolytic and cytotoxic profile, as AMP_2 induced hemolysis in <10% of RBCs at a concentration of 80 µM and resulted in ≈50% cell death at a concentration of 40 µM (Figure 4C,D). In particular, our in vivo experiments revealed that AMP_2 provided complete protection to mice infected with clinical drug‐resistant *E.coli* strains without causing obvious damage. We observed several‐fold changes in some routine blood components, such as white blood cell and lymphocyte counts, between the treatment and control groups (Figure S4, Supporting Information); however, it is commonly assumed that for murine blood components, such variations often fall within the normal physiological range. Thus, AMP_2 holds great promise as an antimicrobial candidate and warrants further investigation.

Although the toxicity of most known AMPs remains inadequately investigated, it is essential to recognize that the antibacterial efficacy of the AMPs generated using ProT‐Diff does not significantly surpass that of the natural AMPs (Figure 4B). To date, powerful deep learning methods have effectively solved the sequence‐structure relationships of proteins with atomic accuracy,^[^ ^24^, ^56^, ^57^ ^]^ enabling researchers to generate idealized protein structures.^[^ ^58^, ^59^, ^60^, ^61^, ^62^ ^]^ However, the de novo design of new proteins or peptides with significantly improved functionality compared to their natural counterparts remains a major challenge. Since the quality of the generated content from deep generative models relies extensively on the quality of the input data, we believe a major obstacle for generative AI is the lack of sufficient training data. For the design of AMPs, there is a persistent goal of creating AMPs characterized by exceptionally low MIC values, minimal toxicity, and other excellent performance characteristics. We believe that this goal can be achieved through generative AI, with the increasing amount and quality of training data in the future.

In this study, we introduce ProT‐Diff and demonstrate its potential through a proof‐of‐concept application in generating AMPs. Notably, ProT‐Diff is not constrained by sequence length and can be readily adapted to generate a wide range of peptides and proteins beyond AMPs. However, the interpretability of ProT‐Diff remains an unresolved issue, as understanding large language and diffusion models continues to be a significant challenge despite their revolutionary impact on AI‐generated content (AIGC). In the field of generative AI, ongoing efforts are focused on developing robust, transparent, and user‐friendly methods to enhance model interpretability. In addition, future theoretical and experimental advancements will be crucial in addressing the issues of model interpretability.

## 4 Conclusion

We present ProT‐Diff, a user‐friendly, cost‐effective, and highly efficient strategy for de novo generation of AMPs with arbitrary lengths from scratch. By integrating a pretrained protein language model with a diffusion model, ProT‐Diff eliminates the need for prior knowledge of peptide structures or sequence alignments. It also reduces GPU memory requirements, training time, and data requirements, thus empowering users, including those with limited expertise in structural biology and computer science. Using ProT‐Diff, we successfully identified several unnatural AMPs with promising performance, highlighting the potential of AMP_2 as a broad‐spectrum therapeutic candidate. Beyond its immediate application in AMP development, this strategy can also facilitate the creation of other peptide‐based drug candidates in the future as well as proteins with tailored characteristics. The versatility demonstrated by our approach underscores its potential impact on advancing diverse aspects of peptide and protein engineering.

## 5 Experimental Section

### Dataset

The known AMP dataset was collected from 4 public AMP databases: CAMPR4 (Collection of Anti‐Microbial Peptides)^[^ ^34^ ^]^ (http://www.camp.bicnirrh.res.in/), ADAM (A Database of Anti‐Microbial peptides)^[^ ^35^ ^]^ (http://bioinformatics.cs.ntou.edu.tw/adam/), APD3 (The Antimicrobial Peptide Database)^[^ ^36^ ^]^ (http://aps.unmc.edu), and GRAMPA (Giant Repository of AMP Activities)^[^ ^37^ ^]^ (https://github.com/zswitten/Antimicrobial‐Peptides). The AMP records were screened based on the following criteria: 1) labeled as antibacterial, antifungal, antiviral, and antimicrobial; 2) have a length of 5 to 48 amino acids; 3) only include capital letters, excluding “U, Z, O, B, J” residues. A total of 17456 known AMPs were identified by combining these four databases and removing duplicate sequences.

The truncated UniProtKB‐reviewed protein dataset was created by truncating sequences from the UniProtKB‐reviewed protein database based on the length distribution of the known AMP dataset. A total of 567834 peptide sequences were collected after removing duplicates. Non‐AMPs containing 58775 peptides were collected from a previously published dataset.^[^ ^13^ ^]^ The training and test sets for the various training tasks were consistently divided at a ratio of 8:2.

### The Overview of the ProT‐Diff Generation Model

First, the peptide sequences were embedded in the training and test sets into tensors of a fixed shape using a pretrained language model for proteins. Peptide embedding features were utilized to independently train a diffusion process in continuous space, which enabled to sample from the trained diffusion process to obtain novel peptide features in the embedding space. Subsequently, the decoder of the pretrained language model was employed to decode the generated peptide features and retrieve amino acid sequences. From the generated peptide sequences, only those predicted as AMPs by the AMP classifier and adhering to predetermined physicochemical property constraints were considered as candidate novel AMPs.

### Peptide Sequence Embedding

The ProtT5‐XL‐UniRef50 encoder of pretrained protein language models was selected from ProtTrans to generate embeddings for the peptide sequences.^[^ ^26^ ^]^ The resulting residue embeddings had a shape of (peptide length, 1024) and were padded with zeros to a shape of (48, 1024) before being input into the subsequent diffusion process.

### Training the Diffusion Process

The training procedure (Figure S6A, Supporting Information) for the diffusion process followed the diffusion‐LM framework.^[^ ^32^ ^]^ The diffusion model was trained directly on known AMPs combined with non‐AMPs or by the pretrain‐finetune approach. For the latter scenario, first pretrained the diffusion process on the truncated UniProtKB‐reviewed protein dataset containing 567834 peptides combined with 17456 known AMPs, and then finetuned the diffusion process on the known AMPs dataset. The sample weights of UniProtKB peptides and the known AMPs during pretraining were set to 0.52 and 16.78, respectively, according to the size of the datasets.

The number of diffusion steps during training was set to 2000. The Trans‐UNet architecture was employed to predict **x**_0_, and the noise schedule was set to *sqrt (square‐root)* schedule proposed by Diffusion‐LM.^[^ ^32^ ^]^ The total number of parameters in the diffusion model is 20706816.

### Sampling for Peptide Features

A DDPM sampler was used to sample peptide features in the embedding space. The diffusion steps of the generative diffusion process were downsampled from 2000 steps to 200 steps according to Diffusion‐LM.^[^ ^32^ ^]^ The noise in each diffusion step was sampled from either a normal distribution, by default, or a uniform distribution. The sampling algorithm is shown in Figure S6B (Supporting Information).

### Decoding the Generated Features

The generated peptide residue embeddings were wrapped as encoder outputs and passed to a ProtT5‐XL‐UniRef50 decoder to reconstruct the amino acid sequences.

### AMP Classifier

The AMP classifier used to distinguish AMPs from non‐AMPs was implemented as a simple MLP with three fully connected layers. The classifier model reads the peptide residue embeddings of ProtT5‐XL‐UniRef50 padded to the shape (48, 1024) as input and outputs the classification results. The classifier was trained using the residue embeddings of known AMPs and non‐AMPs with binary labels. The positive training set of known AMPs was collected from the CAMP, ADAM, and APD3 databases, and the negative training set of non‐AMPs was obtained from a previous study on c_AMP‐prediction.^[^ ^13^ ^]^ The dropout rate of each hidden layer was set to 0.2 and the L2 regularization factor was set to 0.001.

### AMP MIC Predictor

The architecture of the AMP MIC predictor is identical to that of the AMP classifier except for the activation function used in the output layer. The MIC predictor was a regression model trained with the log MIC values of known AMPs from the GRAMPA database.^[^ ^37^ ^]^ For AMPs with multiple target measurements, first took the geometric mean of all MIC values and then took the log to obtain the final log MIC value.

### Pre‐Screening for Candidate Novel AMPs

The generated set of AMP sequences was sieved using the following filters: i) removing duplicates; ii) eliminating sequences already present in the known AMPs set; iii) discarding sequences predicted as non‐AMPs by the AMP classifier; iv) excluding sequences containing more than six tandem repeat amino acids; v) removing sequences with a non‐positive charge; and vi) excluding sequences with a proportion of lysine (K) and arginine (R) residues exceeding 40%. The remaining generated AMPs that met all these constraints were regarded as candidate novel AMPs and used for further experimental validation.

Of the 50 selected candidate peptides synthesized, AMP_1–20 was produced using the first training approach, whereas AMP_21–50 was produced using the second training approach.

### Physicochemical Properties and Structures Prediction

The physicochemical properties of generated peptides are calculated by R package “Peptides”.^[^ ^63^ ^]^ Secondary and tertiary structures of the candidate AMPs were predicted using SABLE^[^ ^49^ ^]^ and AlphaFold2,^[^ ^48^ ^]^ respectively.

### Peptide Synthesis

Peptides were prepared by solid‐phase synthesis with a purity of >90% by Genscript Biotech Corporation (Nanjing, China). The net peptide content was quantified by analyzing the nitrogen content. Peptides such as trifluoroacetic acid (TFA) salts were used in the antimicrobial activity test. The TFA in the peptides was removed by replacement with acetate for the determination of the minimal inhibition concentration (MIC) and animal tests.

### Bacterial Strains

Gram‐negative bacteria include *E. coli* DH5α, *E. coli* O157: H7 CICC 21530, *S. typhimurium* CICC 21484, *A. baumannii* AB6, and *P. aeruginosa* ATCC 15442. Gram‐positive bacteria included *S. aureus* ATCC 33591, Methicillin‐resistant MRSA ATCC 43300, *B. subtilis* ATCC 9372. *L. sphaericus* CCM 2177. Two *E. coli* clinical isolates with *mgrB* mutations from Tongji Hospital (Wuhan, China) were used in this study, namely *E. coli mgrB* mutant 2^#^ and *E. coli mgrB* mutant 23^#^.

### Antimicrobial Activity Test

The antimicrobial activity test was performed as described previously.^[^ ^64^ ^]^ Briefly, the procedure involved the following steps.

First, the tested bacteria were streaked on Luriae–Bertani (LB) agar medium and incubated at 37 °C overnight. Individual colonies were selected from the agar plates and transferred to Mueller–Hinton broth (MHB) for further cultivation. The culture was shaken at 160 rpm at 37 °C overnight. Subsequently, the bacterial suspension was transferred to fresh MHB at a ratio of 1:100 and incubated at 37 °C. When the optical density at 600 nm (OD_600_) of bacterial suspension reached 0.6–0.8, the bacterial suspension was further diluted with MHB to OD_600_ of ≈0.1.

To prepare the antimicrobial peptide solution, AMP was dissolved in sterile water or dimethyl sulfoxide at a concentration of 10 mg mL^−1^. This solution was further diluted with the MHB medium to obtain the desired concentration for testing.

The antibacterial activity tests were conducted in 96‐well plates, and five experimental groups were established as follows: 1) blank control group, MHB solution; 2) without AMPs group, 100 µL of bacterial solution and 100 uL of MHB; 3) AMPs experiment group 1 (low concentration of AMPs, 50 µg mL^−1^), 100 µL of bacterial solution, and 100 uL of 100 µg mL^−1^ AMP; 4) AMPs experiment group 2 (high concentration of AMPs, 150 µg mL^−1^), 100 µL of bacterial solution and 100 uL of 300 µg mL^−1^ AMP; 5) low concentration of AMPs control, 100 µL of MHB solution and 100 uL of 100 µg mL^−1^ AMP; 6) high concentration of AMPs control, 100 µL of MHB solution and 100 uL of 300 µg mL^−1^ AMP. Before and after incubation at 37 °C for 16 h, the OD_600_ value of each well was measured, namely OD_600 (0 h)_ and OD_600 (16 h)_. The relative OD was calculated as AMPs experiment group △OD_600_/ without AMPs group △OD_600_.

(1) $$\begin{array}{ccc} \Delta OD_{600} & = & OD_{600\left(16\;h\right)}-\;\mathrm{control}\;OD_{600\left(16\;h\right)}\\ & & -\left(OD_{600\left(0\;h\right)}-\mathrm{control}\;OD_{600\left(0\;h\right)}\right) \end{array}$$

For the MIC determination of selected AMPs, 100 ul bacterial culture with an OD_600_ value of ≈0.1 was incubated with AMPs at concentrations ranging from 0 to 100 µm at 37 °C for 15–18 h. Herein, the MIC was determined as the minimum concentration of AMPs at which no detectable bacterial growth was observed.

All experiments were conducted with a minimum of four independent replicates. Student's t‐test was used to compare the means between the experimental and control groups (two‐sided).

### TEM Measurement

Transmission Electron Microscopy (TEM) was employed to assess the cell membrane damage induced by AMPs. First, 50 µL of *E. coli* DH5α culture with an OD_600_ of ≈0.1 were mixed with 50 µL of AMPs solution (at 1× MIC) in a 96‐well plate. This mixture was incubated at 37 °C for 2, 4, 8, and 16 h. Following the incubation period, the bacteria were pelleted by centrifugation at 6,000 rpm for 5 min and fixed with 2.5% (vol/vol) glutaraldehyde in phosphate buffer (PB, 0.1 m, pH 7.4). Subsequently, the cells were postfixed with 1% (wt/vol) osmium tetraoxide in PB for 2 h at 4 °C, and were dehydrated through a graded ethanol series (30%, 50%, 70%, 80%, 90%, 100%, with each step lasting 7 min) before being put in pure acetone by two 10‐min steps. Following dehydration, the samples were subjected to infiltration using progressively mixed combinations of acetone and SPI‐PON812 resin (composed of 16.2 g SPI‐PON812, 10 g DDSA, and 8.9 g NMA) in ratios of 3:1, 1:1, and 1:3. The infiltration medium was then replaced with pure resin. Finally, the cells were embedded in pure resin containing 1.5% BDMA and polymerized for 12 h at 45 °C, followed by an additional 48 h polymerization at 60 °C. Ultrathin sections (70 nm thick) were obtained using a microtome (Leica EM UC6), double‐stained with uranyl acetate and lead citrate, and examined using a transmission electron microscope (FEI Tecnai Spirit 120 kV).

### Hemolytic Activity Test

The hemolytic activity of the AMPs was assessed using red blood cells (RBCs) from rabbits. Initially, fresh RBCs were washed three times with phosphate‐buffered saline (PBS) by centrifugation for 15 min at 1,000 g until the supernatant became clear. Subsequently, RBCs were resuspended in PBS to a final erythrocyte concentration of 4% (v/v). Next, 100 µL suspension of RBCs was incubated with 100 µL of AMPs solution at various concentrations at 37 °C for 1 h in 96‐cell cell plates. Following incubation, the supernatant was collected by centrifugation for 15 min at 1000 × g and its absorbance was measured at 540 nm. For reference, hemolysis of RBCs in PBS was designated as representing zero hemolysis, whereas hemolysis of RBCs in 0.1% (w/v) Triton X‐100 was considered 100% hemolysis. The percentage of hemolysis was calculated using the following equation:cen

(2) $$\mathrm{Hemolysis}\left(\%\right)=\frac{OD_{540}\;\mathrm{sample}\;-\;OD_{540}\mathrm{zero}\;\mathrm{lysis}}{OD_{540}100\%\;\mathrm{lysis}\;-\;OD_{540}\;\mathrm{zero}\;\mathrm{lysis}}\times 100\%$$

### Cytotoxicity Against Mammalian Cells

Cell Counting Kit 8 (CCK‐8) assay was used to assess the cytotoxicity of the AMPs against mammalian cells. Specifically, ≈4000 293T cells were seeded per well in a 96‐well cell culture plate (Corning, US), and were incubated at 37 °C with 5% CO_2_ for 24 h. Subsequently, the culture medium was replaced by different concentrations of AMPs solution diluted with the medium. The cells were co‐incubated with AMPs solution for an additional 24 h at 37 °C. And then, 10 µl of the CCK‐8 reagent (Beijing Lablead Biotech, China) was added to each well for 2 h of incubation at 37 °C. Finally, OD at 450 nm was measured using an imaging multimode microplate reader (Cytation 3, BioTek, US). Cell viability was defined as the percentage of each concentration relative to the control. Six replicates were used for the CCK‐8 assay.

### Murine Acute Peritonitis Model

Female BALB/c mice weighing ≈18 g and aged 5–6 weeks were used to build a lethal murine acute peritonitis model according to previously established protocols.^[^ ^43^, ^44^, ^45^ ^]^ The mice were subjected to intraperitoneal injections with *E. colimgrB* mutant 23^#^suspension (0.2 mL per mouse) at concentration of 5×10^5^ CFU mL^−1^, 5×10^6^ CFU mL^−1^, 5×10^7^ CFU mL^−1^, respectively. After infection, mice were observed for 24 h to determine the mortality rate. Each group comprised of eight mice.

To assess the impact of AMP_2 on acute peritonitis, female BALB/c mice were first inoculated with an *E. coli mgrB* mutant 23^#^ suspension at a concentration of 5×10^5^ CFU mL^−1^, which resulted in a mortality rate of 90–100% within 24 h. Subsequently, the mice were administered varying doses of AMP_2 (0.125, 0.25, 0.5, 1, 2, and 4 mg kg^−1^) immediately after infection. As controls, the infected mice received intraperitoneal injections of sterile PBS or underwent penicillin treatment (0.2 mL per mouse). The single dose of penicillin administered was 50000 units per kg, and the mice received antibiotic treatment every 12 h for 3 d. The behavior and survival of the animals were monitored over a 7‐day period. Blood samples were collected from the mice before euthanasia for routine blood tests. The experiments were conducted in groups of eight mice.

All animal experiments were conducted using ABSL‐2. The animal protocol used in this study was approved by the Animal Experimentation Ethics Committee of the National Vaccine & Serum Institute (NVSI) of Sinopharm (NVSI‐RCD‐JSDW‐SER‐2023025) according to China's Guidelines on Welfare and Ethical Review for Laboratory Animals.

## Conflict of Interest

The authors declare no conflict of interest.

## Supporting information



Supporting Information

## Data Availability

The data that support the findings of this study are available from the corresponding author upon reasonable request.
