# Supplementary material for: ProT‐Diff: A Modularized and Efficient Strategy for De Novo Generation of Antimicrobial Peptide Sequences by Integrating Protein Language and Diffusion Models
Source: Adv Sci (Weinh). 2024 Sep 25;11(43):2406305. doi: 10.1002/advs.202406305 (PMC11578372; doi:10.1002/advs.202406305)
Supplement: Supplementary file 1 — Supporting Information [file ADVS-11-2406305-s001.docx]

Supporting Information

ProT-Diff: A Modularized and Efficient Strategy for de novo Generation of Antimicrobial Peptide Sequences by Integrating Protein Language and Diffusion Models

*Xue-Fei Wang^2^, Jing-Ya Tang^1,4^, Jing Sun^1,5^, Sonam Dorje^1,4^, Tian-Qi Sun^2^, Bo Peng^1,4^, Xu-Wo Ji^2^, Zhe Li^2^*, Xian-En Zhang^1,3^*, Dian-Bing Wang^1^**


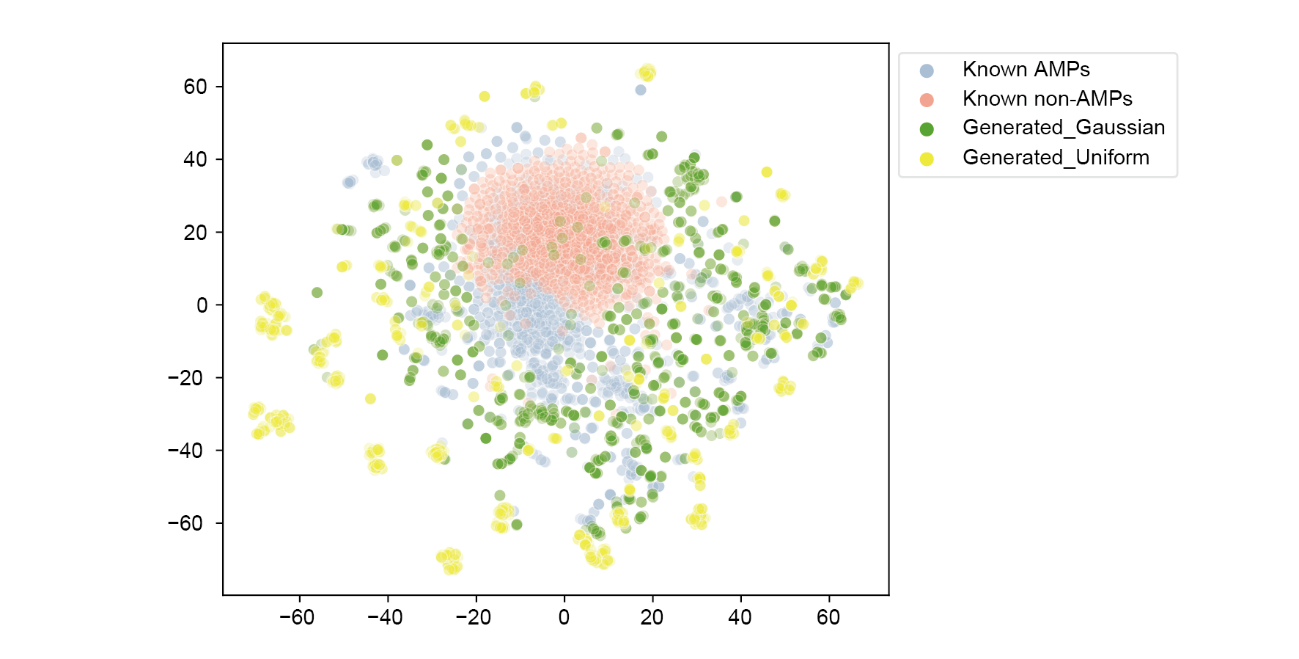


**Figure S1. Comparison of generation diversity using different noise distribution during sampling from the diffusion model.** The t-SNE projection displays the peptide embeddings of known AMPs (blue) and non-AMPs (red) in the training set, as well as the generated peptides resulting from the sampling process with Gaussian noise distribution (green) and Uniform noise distribution (yellow). n=1,000 for each group.


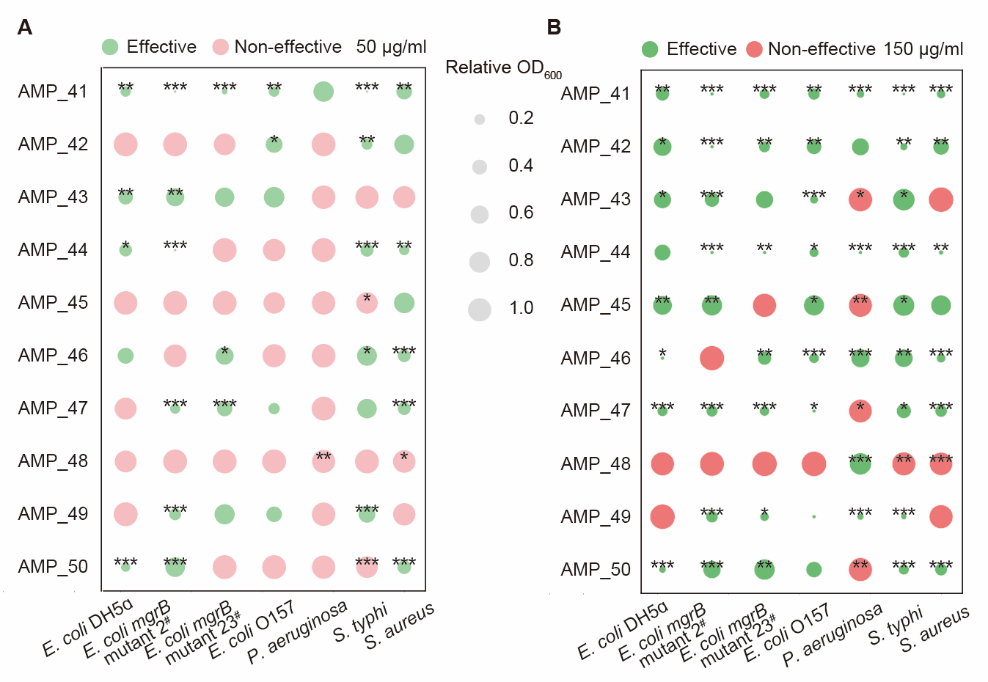


**Figure S2. *In vitro* evaluation of antimicrobial activity for generated candidate AMPs with relatively low sequence identity.** The antimicrobial activities of the 10 candidate AMPs with relatively low sequence identity to known AMPs were assessed at concentrations of 50 μg/mL (**A**) and 150 μg/mL (**B**) against multiple bacteria in liquid medium. The relative OD_600_ of the experimental and control groups were compared, with an effectiveness threshold of 0.8. Any relative OD_600_ value below 0.8 was considered effective. The means between the experimental and control groups were compared using a two-sided Student’s t-test (*, **, and *** indicates 0.01 <*P* ≤ 0.05, 0.001 <*P* ≤ 0.01, and *P* ≤ 0.001, respectively). n=4 for each group.


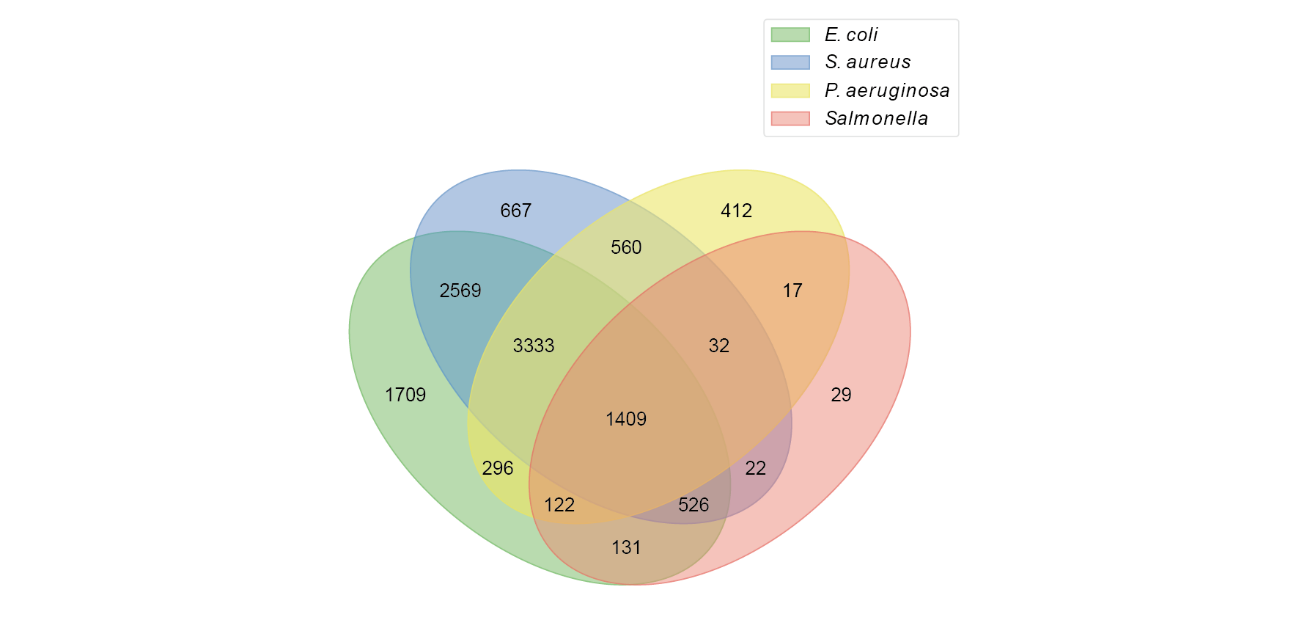


**Figure S3. The presence of species target labels in known AMPs.** The overlap of known AMPs targeting *E. coli*, *S. aureus*, *P. aeruginosa*, and *Salmonella*.


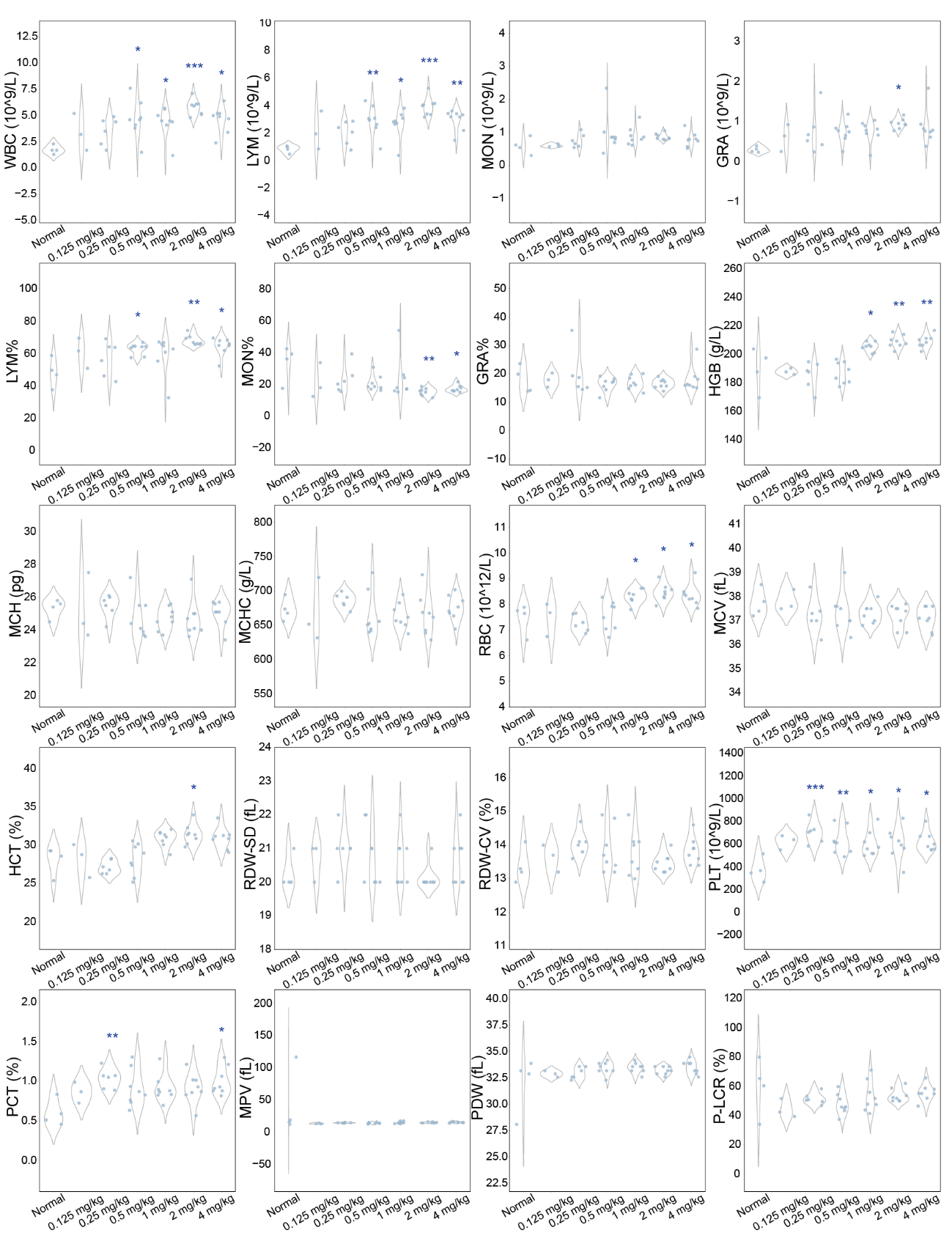


**Figure S4. Routine blood analysis of mice following treatment with AMP_2 during infection.** WBC, white blood cells; LYM, lymphocytes; MON, monocytes; GRA, granulocytes; HGB, hemoglobin; MCH, mean corpuscular hemoglobin; MCHC, mean corpuscular hemoglobin concentration; RBC, red blood cells; MCV, mean corpuscular volume; HCT, hematocrit; RDW-CV, red cell distribution width reported statistically as coefficient of variation; RDW-SD, RDW reported statistically as coefficient of standard deviation; PLT, platelet; PCT, procalcitonin; MPV, mean platelet volume; PDW, platelet distribution width; P-LCR, platelet-large cell ratio. The means between the experimental and normal groups were compared using a two-sided Student’s t-test (*, **, and *** indicates 0.01 <*P* ≤ 0.05, 0.001 <*P* ≤ 0.01, and *P* ≤ 0.001, respectively).

**
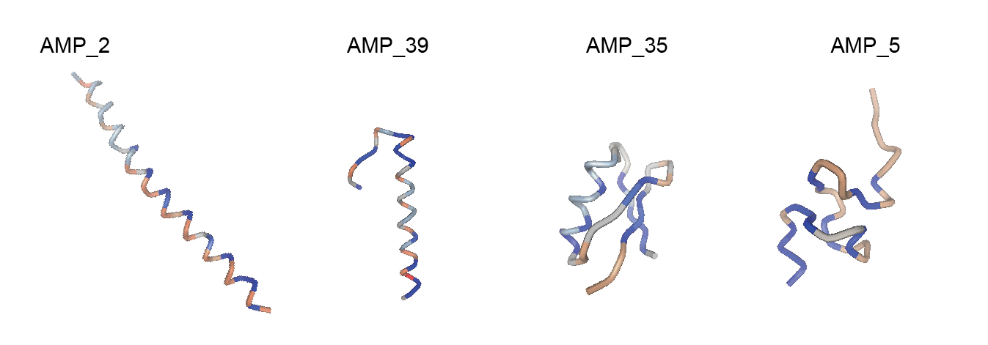
**

**Figure S5. Typical predicted structures of candidate AMPs.** The four major types of tertiary structures of candidate AMPs predicted by AlphaFold2. The backbone is colored by amino charge.

| **A** Diffusion Training | |
| --- | --- |
| Require: Peptide sequences $\mathbf{w}$, AMP binary label $y$, Peptide embeddings $\mathcal{D}$,  Original data sample $\mathbf{x}_{\mathbf{0}}$, Latent variables $\mathbf{x}_{\boldsymbol{1}}\boldsymbol{,\ldots,}\mathbf{x}_{\boldsymbol{T}}$,  Protein sequence encoder $E\text{()}$, Denoising model $\boldsymbol{\mu}_{\boldsymbol{\theta}}\text{()}$,  Timesteps $T$, Noise $\boldsymbol{\epsilon}$, Noise schedule $\bar{\alpha_{t}}$, Learning rate $\text{η}$ | |
| 1. **repeat** |  |
| 1. $\mathbf{x}_{\mathbf{0}}\boldsymbol{=}E\text{(}\mathbf{w}\text{)}\boldsymbol{\sim}\mathcal{D}$ | Encode sampled data |
| 1. $t \sim\mathcal{U}\text{(}\{1,\ldots, T\}\text{)}$ | Sample timestep |
| 1. $\boldsymbol{\epsilon}\mathcal{\sim N}\text{(}0,\mathbf{I}\text{)}$ | Sample noise |
| 1. $\mathbf{x}_{\boldsymbol{t}}\boldsymbol{=}\sqrt{\bar{\alpha}_{t}}\mathbf{x}_{\mathbf{0}}\boldsymbol{+}\sqrt{1\boldsymbol{-}\bar{\alpha}_{t}}\boldsymbol{\epsilon}$ | Compute latent variables |
| 1. $\mathcal{L=}\left\Vert\boldsymbol{\mu}_{\boldsymbol{\theta}}\text{(}\mathbf{x}_{\boldsymbol{t}},t\text{)}-\mathbf{x}_{\boldsymbol{0}} \right\Vert_{\text{2}}^{2}$ | Compute loss |
| 1. $\theta\text{=}\theta-\text{η}\nabla_{\theta}\mathcal{L}$ | Update parameters |
| 1. **until** converged |  |

| **B** Diffusion Sampling | |
| --- | --- |
| Require: Estimated original data ${\tilde{\mathbf{x}}}_{\mathbf{0}}$, Latent variables $\mathbf{x}_{\text{1}}\boldsymbol{,\ldots,}\mathbf{x}_{\boldsymbol{T}}$,  Denoising model $\boldsymbol{\mu}_{\boldsymbol{\theta}}\text{()}$, Timesteps $T$, Noise $\boldsymbol{\epsilon}$, Noise schedule $\bar{\alpha_{t}}$,  Protein sequence decoder$\text{ }D\text{()}$ | |
| 1. $\mathbf{x}_{\boldsymbol{T}}\mathcal{\sim N}\text{(}0,\mathbf{I}\text{)}$ | Sample the initial latent variable |
| 1. **for** $t=T,\ldots,1$ **do** |  |
| 1. ${\tilde{\mathbf{x}}}_{\mathbf{0}}\boldsymbol{=}\boldsymbol{\mu}_{\boldsymbol{\theta}}\text{(}\mathbf{x}_{\boldsymbol{t}},t\text{)}$ | Estimate original data $\mathbf{x}_{\mathbf{0}}$ |
| 1. **if** $t=1$ **then** |  |
| 1. **return** ${\tilde{\mathbf{x}}}_{\mathbf{0}}$ | Get generated data |
| 1. **end if** |  |
| 1. $\boldsymbol{\epsilon}\mathcal{\sim N}\text{(}0, \mathbf{I}\text{)}\mathrm{or}\mathcal{U}\text{(}-3, 3\text{)}$ | Sample noise |
| 1. $\mathbf{x}_{\boldsymbol{t-1}}\boldsymbol{=}\sqrt{\bar{\alpha}_{t-1}}{\tilde{\mathbf{x}}}_{\mathbf{0}}\boldsymbol{+}\sqrt{1\boldsymbol{-}\bar{\alpha}_{t-1}}\boldsymbol{\epsilon}$ | Compute the next latent variable |
| 1. **end for** |  |
| 1. $\text{w}\boldsymbol{'=}D\text{(}{\tilde{\mathbf{x}}}_{\mathbf{0}}\text{)}$ | Decode generated data |

Figure S6. Diffusion training (A) and sampling (B) algorithms.

Table S1. Basic information of the 50 selected generated AMPs.

| No. | Sequence | Molecular Weight (g/mol)^a)^ | Length | Net charge (Dawson) | pI (Dawson) | Hydrophobicity index (Eisenberg) | Hydrophobic moment | Predicted structure^b)^ | Closest known AMPs | Identity | Concentrate at 50 ug/mL (uM) | Concentrate at 150 ug/mL (uM) |
| --- | --- | --- | --- | --- | --- | --- | --- | --- | --- | --- | --- | --- |
| AMP_1 | FLLLLAAILAKVLCCICKKCKKCKK | 2794.91 | 25 | 6.70 | 9.81 | 0.17 | 0.57 | 68.0% α-helix (1 helix) | CAMPSQ3235 | 55.00 | 17.89 | 53.67 |
| AMP_2 | KWKFFKKIRKVGKKIRKVIKKIGKAVGAAAQAAGQAANA | 4208.42 | 39 | 12.94 | 12.19 | -0.12 | 0.97 | 89.7% α-helix (1 helix) | ADAM_6711 | 60.61 | 11.88 | 35.64 |
| AMP_3 | GLFKKIKKGIKNGIKNVAKEVAKEVAKEVAKEVACKVAKKCS | 4513.79 | 42 | 8.85 | 10.44 | -0.13 | 0.82 | 88.1% α-helix (1 helix) 2.4% β-strand/bridge (1 strand/bridge) | CAMPSQ3602 | 36.36 | 11.08 | 33.23 |
| AMP_4 | RVKRVWRVVRRVIRVVRAVYRAI | 2905.75 | 23 | 8.94 | 12.48 | -0.35 | 1.21 | 87.0% α-helix (1 helix) | CAMPSQ18481 | 52.17 | 17.21 | 51.62 |
| AMP_5 | LFCFLRCCFSSCCHRHCCRRHCCRRHCCRRWRRRRRR | 4915.09 | 37 | 13.78 | 11.23 | -0.75 | 0.63 | 21.6% α-helix (3 helices) 2.7% β-strand/bridge (1 strand/bridge) | ADAM_6614 | 50.00 | 10.17 | 30.52 |
| AMP_6 | ESLSSSCCRLCRLCCCCR | 2024.53 | 18 | 1.61 | 7.81 | -0.21 | 0.60 | 55.6% α-helix (1 helix) | CAMPSQ9314 | 37.50 | 24.70 | 74.09 |
| AMP_7 | LVRGCWTKCCPKSCCVRCC | 2147.79 | 19 | 3.61 | 8.28 | -0.09 | 0.30 | 21.1% α-helix (1 helix) | CAMPSQ329 | 72.22 | 23.28 | 69.84 |
| AMP_8 | FFFHIIHHIFHLIRRLLHRIRRRRRRHR | 3895.91 | 28 | 10.49 | 12.95 | -0.46 | 1.00 | 75.0% α-helix (1 helix) 10.7% β-strand/bridge (1 strand/bridge) | CAMPSQ22815 | 51.85 | 12.83 | 38.50 |
| AMP_9 | GIGKGIKKALKGLKGLFKGLCKGC | 2417.27 | 24 | 6.84 | 10.63 | 0.09 | 0.79 | 66.7% α-helix (1 helix) | CAMPSQ1163 | 50.00 | 20.68 | 62.05 |
| AMP_10 | ATCDLLSSSSSSAACAAACAAHCKRGGYCKKKRCCRRRK | 4108.97 | 39 | 8.70 | 9.82 | -0.31 | 0.49 | 23.1% α-helix (1 helix) 2.6% β-strand/bridge (1 strand/bridge) | CAMPSQ8345 | 55.00 | 12.17 | 36.51 |
| AMP_11 | QRAVRRIYRIIRRIPRILRRIPRAIRAARRASQALWRAKSQ | 5049.32 | 41 | 14.94 | 12.78 | -0.52 | 1.26 | 90.2% α-helix (1 helix) | ADAM_3793 | 30.00 | 9.90 | 29.71 |
| AMP_12 | RRFFFFFFRFR | 1672.05 | 11 | 3.94 | 12.48 | -0.16 | 0.51 | Unknown | ADAM_3754 | 36.36 | 29.90 | 89.71 |
| AMP_13 | FVLLVIIIKCKKCKC | 1750.48 | 15 | 3.80 | 9.39 | 0.30 | 0.27 | 20.0% α-helix (1 helix) 20.0% β-strand/bridge (1 strand/bridge) | CAMPSQ536 | 50.00 | 28.56 | 85.69 |
| AMP_14 | FFGVVFKVASKVFSAVFGKV | 2162.75 | 20 | 2.94 | 10.80 | 0.49 | 0.58 | 65.0% α-helix (2 helices) 15.0% β-strand/bridge (1 strand/bridge) | CAMPSQ2788 | 85.00 | 23.12 | 69.36 |
| AMP_15 | GLWKKIKNAAKSAAKAAGKAAK | 2210.8 | 22 | 6.94 | 11.28 | -0.10 | 0.67 | 81.8% α-helix (1 helix) | CAMPSQ1059 | 66.67 | 22.62 | 67.85 |
| AMP_16 | SFFSTFKNAAKNAAKNAAQSALNSLKCKLSKKCK | 3648.45 | 34 | 7.84 | 10.72 | -0.15 | 0.62 | 61.8% α-helix (1 helix) 2.9% β-strand/bridge (1 strand/bridge) | CAMPSQ3557 | 66.67 | 13.70 | 41.11 |
| AMP_17 | GIIDIIKNLFKKIKKIKK | 2139.92 | 18 | 5.94 | 10.90 | -0.07 | 0.87 | 72.2% α-helix (1 helix) 11.1% β-strand/bridge (1 strand/bridge) | CAMPSQ14127 | 64.71 | 23.37 | 70.10 |
| AMP_18 | RFGRFLRKIRRFRRFITTITTTITTTT | 3360.16 | 27 | 7.94 | 12.78 | -0.29 | 1.06 | 40.7% α-helix (1 helix) 7.4% β-strand/bridge (1 strand/bridge) | CAMPSQ1054 | 59.26 | 14.88 | 44.64 |
| AMP_19 | WGWRDIVRGIRRIIRAIAAAL | 2462.11 | 21 | 3.94 | 12.18 | 0.03 | 1.20 | 85.7% α-helix (1 helix) | CAMPSQ23001 | 60.00 | 20.31 | 60.92 |
| AMP_20 | GFLDTFKNAAKNAAKNAAKNAAKNAACKLLSKAC | 3467.21 | 34 | 5.84 | 10.38 | -0.06 | 0.64 | 88.2% α-helix (1 helix) | CAMPSQ705 | 55.88 | 14.42 | 43.26 |
| AMP_21 | GLGSLLGKLLKTLGKTLKKIFGKKRRQQQ | 3238.23 | 29 | 8.94 | 12.14 | -0.21 | 0.78 | 69.0% α-helix (2 helices) | CAMPSQ3382 | 53.57 | 15.44 | 46.32 |
| AMP_22 | KWKLFKKVFKKVLPAVLKVLKTALKALK | 3266.46 | 28 | 9.94 | 11.45 | 0.03 | 0.83 | 92.9% α-helix (1 helix) | CAMPSQ8694 | 64.29 | 15.31 | 45.92 |
| AMP_23 | RIGSILGSILGALKSLLKSIKKRR | 2607.43 | 24 | 6.94 | 12.34 | -0.06 | 0.75 | 79.2% α-helix (1 helix) | ADAM_6360 | 52.17 | 19.18 | 57.53 |
| AMP_24 | FLPAVASIAATILSKICKKCSKKCS | 2610.39 | 25 | 4.80 | 9.94 | 0.15 | 0.64 | 72.0% α-helix (1 helix) | CAMPSQ19697 | 62.50 | 19.15 | 57.46 |
| AMP_25 | GKFSIISKIFRVIRKVFKVVKKVFKAVKKAFKSAKKKN | 4424.82 | 38 | 14.94 | 12.22 | -0.15 | 1.05 | 76.3% α-helix (1 helix) 5.3% β-strand/bridge (1 strand/bridge) | ADAM_2239 | 58.82 | 11.30 | 33.90 |
| AMP_26 | FIRHIWKFLRKLF | 1803.36 | 13 | 4.03 | 12.05 | 0.06 | 0.99 | 84.6% α-helix (1 helix) | CAMPSQ14104 | 61.54 | 27.73 | 83.18 |
| AMP_27 | FLPGLLSCVCKIVCTIFCRICRIIC | 2816.8 | 25 | 2.65 | 8.16 | 0.41 | 0.74 | 84.0% α-helix (1 helix) | ADAM_1311 | 54.55 | 17.75 | 53.25 |
| AMP_28 | GWLDKLLKFLKKLLKVIKKILKK | 2793.87 | 23 | 7.94 | 11.04 | -0.03 | 0.89 | 87.0% α-helix (1 helix) | CAMPSQ10244 | 43.48 | 17.90 | 53.69 |
| AMP_29 | GLWSKIKSAGKAAAKAAAKAVTKAVTKAV | 2825.57 | 29 | 6.94 | 11.28 | 0.09 | 0.61 | 87.0% α-helix (1 helix) | ADAM_2796 | 65.52 | 17.70 | 53.09 |
| AMP_30 | FFHHIFRGIRHIFRHIRRIFRHI | 3111.9 | 23 | 6.40 | 12.70 | -0.11 | 1.16 | 91.3% α-helix (1 helix) | CAMPSQ20992 | 63.16 | 16.07 | 48.20 |
| AMP_31 | AKLLKALLKLLKKLA | 1663.36 | 15 | 4.94 | 11.10 | 0.12 | 0.78 | 93.3% α-helix (1 helix) | CAMPSQ14610 | 57.14 | 30.06 | 90.18 |
| AMP_32 | GFFGLLAKAAKHAAKHIHRRRRRHRHR | 3284.04 | 27 | 10.39 | 12.79 | -0.53 | 0.72 | 74.1% α-helix (1 helix) | CAMPSQ10520 | 56.00 | 15.23 | 45.68 |
| AMP_33 | GLLSALRSLLSLLSHIIKHIKK | 2440.22 | 22 | 4.12 | 11.53 | 0.19 | 0.72 | 90.9% α-helix (1 helix) | AP02740 | 59.09 | 20.49 | 61.47 |
| AMP_34 | GWLRKAAKSARKAVKKAAKAVKKAFKAAKHAIK | 3560.58 | 33 | 13.03 | 12.19 | -0.25 | 0.83 | 93.9% α-helix (1 helix) | CAMPSQ489 | 50.00 | 14.04 | 42.13 |
| AMP_35 | GVCVALSGSSSAAACAAACALLGRRGGYCRGRGGCVCRRR | 3902.75 | 40 | 6.65 | 9.65 | -0.03 | 0.36 | 25.0% α-helix (1 helix) 20.0% β-strand/bridge (2 strands/bridges) | CAMPSQ2745 | 56.41 | 12.81 | 38.43 |
| AMP_36 | GLLKFLKKLAKFFAALFAAIIKK | 2579.48 | 23 | 5.94 | 11.20 | 0.32 | 0.77 | 87.0% α-helix (1 helix) | CAMPSQ23240 | 45.00 | 19.38 | 58.15 |
| AMP_37 | RRRRVIIVVVVCR | 1623.16 | 13 | 4.89 | 12.18 | -0.32 | 0.21 | 69.2% β-strand/bridge (1 strand/bridge) | CAMPSQ9675 | 61.54 | 30.80 | 92.41 |
| AMP_38 | MAARLARLAARLARLALRLLR | 2375.12 | 21 | 5.94 | 12.70 | -0.13 | 1.03 | 95.2% α-helix (1 helix) | CAMPSQ18502 | 52.63 | 21.05 | 63.15 |
| AMP_39 | SILDKIKNAVKGAAKGAAKGLLCKLLPKSCCKSC | 3460.5 | 34 | 6.75 | 10.05 | 0.02 | 0.68 | 73.5% α-helix (2 helices) | ADAM_6848 | 61.77 | 14.45 | 43.35 |
| AMP_40 | VQYRIRIRIVIRK | 1712.25 | 13 | 4.94 | 12.02 | -0.35 | 0.41 | 7.7% α-helix (1 helix) 76.9% β-strand/bridge (2 strands/bridges) | CAMPSQ9402 | 55.56 | 29.20 | 87.60 |
| AMP_41 | GLLKDTLKAAMKAVKAAIKAAIK | 2353.12 | 23 | 4.94 | 10.80 | 0.11 | 0.61 | 87.0% α-helix (1 helix) | CAMPSQ3665 | 40.91 | 21.25 | 63.75 |
| AMP_42 | GLLKDTLKKLLKKLKKAVKAVIN | 2562.47 | 23 | 6.94 | 10.98 | -0.09 | 0.84 | 78.3% α-helix (1 helix) | CAMPSQ23684 | 39.13 | 19.51 | 58.54 |
| AMP_43 | ASHLPSSLSSAASKLASALSKSLSSVLTKLSKK | 3285 | 33 | 5.03 | 11.10 | 0.05 | 0.54 | 75.8% α-helix (1 helix) | CAMPSQ21749 | 42.42 | 15.22 | 45.66 |
| AMP_44 | RLNALLHWLRNWAKKKK | 2174.78 | 17 | 6.03 | 12.09 | -0.35 | 0.65 | 70.6% α-helix (1 helix) | CAMPSQ14602 | 35.71 | 22.99 | 68.97 |
| AMP_45 | GLSRLLSALR | 1084.39 | 10 | 1.94 | 12.00 | -0.01 | 0.62 | NA | NA | NA | 46.11 | 138.33 |
| AMP_46 | GKFSKIFKIIRKVFKVVRKVFKAVKKAFKSAKKASKSA | 4327.64 | 38 | 14.94 | 12.22 | -0.16 | 1.02 | 78.9% α-helix (1 helix) | ADAM_3392 | 26.67 | 11.55 | 34.66 |
| AMP_47 | KWKIFKKIEKIIKKIEKVVKKVAKAAGKAAQAAAAVAS | 4092.32 | 38 | 9.94 | 10.98 | -0.02 | 0.94 | 86.8% α-helix (1 helix) 2.6% β-strand/bridge (1 strand/bridge) | CAMPSQ3474 | 41.67 | 12.22 | 36.65 |
| AMP_48 | GWKGWAKKAGKKAKGAAKAAAKAAAQQ | 2681.31 | 27 | 7.94 | 11.35 | -0.12 | 0.54 | 63.0% α-helix (2 helices) | CAMPSQ24080 | 43.48 | 18.65 | 55.94 |
| AMP_49 | RPAFKRAFRAAFRAARC | 1994.44 | 17 | 5.89 | 12.18 | -0.38 | 0.99 | 82.4% α-helix (1 helix) | AP03154 | 37.50 | 25.07 | 75.21 |
| AMP_50 | TRSSSRAGLGVVVRVLRRLRRR | 2550.17 | 22 | 7.94 | 12.85 | -0.53 | 0.99 | 50.0% α-helix (1 helix) | ADAM_7230 | 33.33 | 19.61 | 58.82 |

^a)^ The physicochemical properties of generated peptides are calculated by R package “Peptides”^[63]^.

^b)^ The secondary and tertiary structures of candidate AMPs are predicted by SABLE^[49]^ and AlphaFold2^[48]^, respectively.

Table S2. Comparison of generated sequences passed *in silico* filters in the two training approaches of the diffusion model.

| In silico filters | Remaining sequence counts | |
| --- | --- | --- |
|  | training once on  AMPs + non-AMPs | pretraining + finetuning |
| Total | 5,000 | 5,000 |
| Drop duplicates | 2,158 | 2,443 |
| Not present in the known AMP set | 2,145 | 2,365 |
| Predicted as AMPs by the AMP classifier | 483 | 2,164 |
| Contain no more than 6 tandam repeat amino acids | 337 | 1,745 |
| Have positive net charge | 316 | 1,548 |
| Have no more than 40% of K + R | 251 | 986 |

Table S3. Comparison of AI-based models used for AMPs discovery.

| Model name | Model type | Architecture | Coupling of sequence representation and generation | Input info | Maxium length of AMP | In silico success rate | Experimental success rate | Reference |
| --- | --- | --- | --- | --- | --- | --- | --- | --- |
| ProT-Diff | Generative | ProtT5, Diffusion | No | AMP and non-AMP sequences | 48 | 986/5,000 | 44/45 | This study |
| A peptide VAE model | Generative | VAE | Yes | AMP sequences | 12 | Undetermined | 9/14 | 15 |
| PepVAE | Generative | VAE | Yes | AMP sequences | 40 | 38/100 | 37/38 | 16 |
| CLaSS | Generative | VAE/WAE | Yes | AMP and non-AMP sequences | 25 | 163/90,000 | 2/20 | 17 |
| HydrAMP | Generative | cVAE | Yes | AMP and non-AMP sequences | 25 | 92/900, 84/900 | 23/24 | 18 |
| PepCVAE | Generative | cVAE | Yes | AMP and non-AMP sequences | 30 | 45/5,000 | Undetermined | 19 |
| AMPGAN v2 | Generative | BiCGAN | Yes | AMP and non-AMP sequences | 32 | 88.36% (n=5,000) | 3/6 | 20 |
| A unified pipeline | Predictive | LSTM, Attention and BERT | Unapplicable | AMP and non-AMP sequences | 50 | Unapplicable | 181/216 | 13 |
| SMEP | Predictive | XGBoost-tab, LSTM | Unapplicable | AMP and non-AMP sequences | 9 | Unapplicable | 10/10 | 14 |
